# Combined lifestyle factors on mortality and cardiovascular disease among cancer survivors: a systematic review and meta-analysis of cohort studies

**DOI:** 10.1007/s00520-024-09049-2

**Published:** 2024-12-02

**Authors:** Chunsu Zhu, Zhiwei Lian, Volker Arndt, Melissa S. Y. Thong

**Affiliations:** 1 Unit of Cancer Survivorship, German Cancer Research Center (DKFZ), Im Neuenheimer Feld 280, 69120 Heidelberg, Germany; 2 Medical Faculty, University of Heidelberg, Heidelberg, Germany

**Keywords:** Lifestyle, Cancer survivor, Mortality, Cardiovascular disease, Systematic review, Meta-analysis

## Abstract

**Purpose:**

Lifestyle factors in cancer survivors are frequently studied individually even though they are often interconnected. This systematic review and meta-analysis investigated the association of combined lifestyle factors on health outcomes among cancer survivors.

**Methods:**

EMBASE, PubMed and Web of Science were searched up to March 2024. Cohort studies examining the associations of at least three combined lifestyle factors with mortality (all-cause/cancer-specific) and cardiovascular disease (CVD) among cancer survivors, were selected. Pooled hazard ratios (pHRs) and 95% confidence intervals (CIs) were estimated using random effects models. Tests for heterogeneity and publication bias were conducted.

**Results:**

Twenty-two studies involving 209,659 survivors with an average follow-up duration ranging from 4.1 to 29.1 years were included. pHRs(95%CI) comparing cancer survivors with the healthiest lifestyles versus those with the least healthy lifestyles were 0.57 (0.51–0.65) for all-cause mortality, 0.70 (0.61–0.80) for cancer-specific mortality, and 0.53 (0.46–0.63) for CVD incidence. These associations were largely consistent across subgroup analyses. Colorectal cancer survivors with the healthiest lifestyle experienced 37% lower all-cause mortality and 25% lower cancer-specific mortality, while breast cancer survivors had a 45% reduction in all-cause mortality. Although studies were limited, significant associations for all-cause mortality were observed among lung, liver, nasopharyngeal, gastric, kidney, gynecologic cancer survivors. However, no significant relationship between healthy lifestyles and CVD-specific mortality was detected.

**Conclusions:**

Having an overall healthy lifestyle is associated with lower CVD incidence and better survival among cancer survivors. The long-term management of cancer survivors should consider encouragement for a modification of multiple lifestyles.

**Supplementary information:**

The online version contains supplementary material available at 10.1007/s00520-024-09049-2.

## Introduction

Cancer is a major public health issue worldwide, with an estimated 20 million new cancer cases and 10 million cancer deaths in 2022 [1, 2]. Due to advances in treatment and early detection, the survival trends for most cancers are increasing, leading to a rapidly growing population living with a cancer diagnosis [3]. More than 16.9 million citizens were living with a diagnosis of cancer in the United States (US) alone in 2019, and the number is expected to reach more than 22.1 million by 2030 [4]. Although the 5-year survival for all cancers combined has reached 68%, cancer survivors still suffer from excess long-term physical effects (e.g., cardiovascular disease [CVD] [5]) and mortality compared to the general population [4, 6]. Adopting an overall healthy lifestyle was reported to be the “best buy” strategy for preventing premature deaths and cancer in the general population [7, 8]. However, whether the modification of multiple lifestyle factors could improve the prognosis of cancer survivors remains unclear. Previous evidence has shown that some lifestyles (e.g., diet and physical activity) are associated with the survival of cancer survivors [9]. Nevertheless, most of these studies focused on only one or two lifestyles and failed to consider that many lifestyle factors are interconnected and concurrent. In reality, survivors rarely change only one or two aspects of their lifestyle. Thus, investigating lifestyles individually may overlook the cumulative effects that these lifestyles have on the prognosis of cancer survivors, and treating lifestyle factors in combination might provide better insight into this relationship. Unfortunately, to our knowledge, to date, no rigorous randomized controlled trials (RCTs) have examined the effects of comprehensive lifestyle interventions on the prognosis and survival of cancer survivors because it seems impractical and costly to recruit a large number of cancer patients and follow them for 5 or 10 years to examine the effect of lifestyle interventions on survival and other long-term health outcomes [10]. Alternatively, evidence from long-term cohort studies is of vital importance for the guidance of clinical practices and public health initiatives. In recent years, there has been a growing number of cohort studies examining the association between combined lifestyles and health outcomes among cancer survivors [11–13]. However, no systematic review and meta-analysis is currently available.

Therefore, the purpose of this systematic review and meta-analysis was to explore the relationships of combined lifestyles with all-cause mortality, cancer-specific mortality, and CVD among cancer survivors. In addition, we assessed whether these associations varied across different characteristics and cancer diagnoses.

## Methods

This review is registered in the International Prospective Register of Systematic Review (CRD42024521538) and was performed and reported according to the guidelines of the Meta-analysis of Observational Studies in Epidemiology (MOOSE) [14].

### Search strategy and study selection

PubMed, EMBASE and Web of Science were searched from inception to March 2024. Various combinations of the following keywords were used: “combined”, “lifestyle”, “mortality”, “cardiovascular disease”, “cancer survivors”, and “cohort study”. Detailed search strategies were provided in the supplementary file (Tables S1-S3). The language was restricted to English. The reference lists of the included studies and relevant reviews were checked manually to identify further pertinent publications.

The study selection was conducted independently by two reviewers (CZ and ZL). Any controversies were resolved by a senior investigator (MT). Prospective cohort studies investigating associations between a combination of at least three lifestyle factors and all-cause mortality, cancer-specific mortality, CVD incidence or CVD mortality among cancer survivors, were included. Protocols, reviews, cross-sectional studies, RCTs, animal experiments, and meeting abstracts were excluded. We also excluded studies focusing on a single lifestyle or a combination of only two lifestyles because we assumed that overall lifestyles could not be comprehensively reflected by only one or two lifestyle factors; studies focusing on general population rather than cancer survivors; studies about the formulation of prediction models; and studies without adequate results.

### Data extraction

Data extraction and quality assessment were performed by CZ and ZL separately, and any disagreements were addressed through discussion with a senior researcher (MT). The following data were extracted: title, first author, publication year, country, average follow-up duration, combined lifestyle factors, adjusted confounders, mean age at diagnosis, sex, cancer diagnosis, outcomes, sample size, and adjusted risk estimates with corresponding 95% confidence intervals (CIs). For publications with missing information, we contacted the corresponding author at least two times to request the relevant data. The Newcastle–Ottawa scale (NOS), which is based on the selection of cohorts (four items), the comparability of cohorts (three items) and the ascertainment of outcomes (three items), was used to evaluate the quality of studies, a sum score of 10 was generated, with higher score indicating better quality [15].

### Statistical analysis

All analyses were conducted using the Metafor package in R (version 4.3.3), and statistical significance was set at a two-sided *P* value < 0.05. Pooled hazard ratios (pHRs) and corresponding 95% CIs were calculated using random effects models, allowing heterogeneity between studies. Risk ratios (RRs) were regarded as interchangeable with HRs. Data were first synthesized by comparing the highest lifestyle score group with the lowest score group, which indicated the differences between the group adopting the healthiest lifestyle pattern and the group adopting the least healthy lifestyle. Then, a linear dose–response meta-analysis was also performed by pooling the studies that reported results of per-unit increase in the score. Forest plots were used to present the effect sizes and corresponding 95% CIs across studies.

*I^2* statistics and *P* values for the Cochran Q test were used to estimate the heterogeneity between studies [16]. To test the sources of heterogeneity, subgroup analyses were further carried out stratified by population characteristics (e.g., cancer type, age, sex) and study characteristics (e.g., country, follow-up duration, different lifestyle scores), as appropriate. Small study bias was examined by Egger’s test and visualization of funnel plots. Finally, leave-one-out analysis was performed to assess each study’s influence on the pooled HRs.

## Results

### Study characteristics

The process of study selection is shown in Supplementary Figure S1. Overall, 22 studies published from 2013 to 2024 were included (17 for all-cause mortality, 10 for cancer-specific mortality, three for CVD incidence, and two for CVD-specific mortality). Their main characteristics are summarized in Table 1. A total of 209,659 cancer survivors aged 6.0 to 78.9 years (age at diagnosis) were included. The average follow-up time ranged from 4.2 to 29.1 years. Most studies were performed in mixed cancer survivors (n = 7), colorectal cancer survivors (n = 6), and breast cancer survivors (n = 5). The other studies were on survivors of prostate cancer (n = 2), ovarian cancer (n = 1), and those treated with hematopoietic cell transplantation (HCT, n = 1). The majority of studies were carried out in adult cancer survivors (n = 21), and only one study involved adult survivors of childhood cancer. The studies were mainly conducted in high-income countries (North America: n = 16, Europe: n = 5). The NOS scores of these studies were all ≥ 6 (Supplementary Table S4). The main causes of low quality included insufficient representative of exposed cohort, no adjustment of baseline health status, and inadequate follow-up duration (10 ≥ years).

**Table 1:** Characteristics of included studies

First author(year)	Country	Sample size	Outcomes	Mean follow-up duration in years	Men (%)	Average age at diagnosis year	Tumor types
Bian (2024) [11]	USA, UK, China	37,095	All-cause mortality Cancer-specific mortality	5	55.9	60 +	Mixed
Barot (2024) [37]	Sweden	1098	All-cause mortality	4.3	52.2	69	CRC
Troeschel (2023) [12]	USA	1964	All-cause mortality Cancer-specific mortality	10	0	61.2	BC
Peng (2023) [13]	UK	13,348	CVD incidence	8.01	0	52.4	BC
Langlais (2023) [38]	USA	2056	Cancer-specific mortality	6.4	100	64.5	PC
Ergas (2023) [39]	USA	3658	All-cause mortality	up to 10	0	59.7	BC
Cannioto (2023) [18]	USA	1340	All-cause mortality	7.7	0	51.3	BC
Liu (2022) [40]	USA	3145	All-cause mortality Cancer-specific mortality	6.3	43.1	62.7	Mixed
Graff (2022) [41]	USA	4518	Cancer-specific mortality	10.2	100	69 +	OC
Zutphen (2021) [20]	Netherlands	1425	All-cause mortality	4.4	57.6	66	CRC
Sun (2021) [42]	China	46,120	All-cause mortality	4.3	45.7	57	Mixed
Song (2021) [19]	USA	1491	All-cause mortality Cancer-specific mortality	7.92	57.6	68.5	CRC
Cao (2021) [43]	UK	35,564	CVD incidence	up to 15	33	59.3	Mixed
Minlikeeva (2019) [44]	High income countries	7022	All-cause mortality	5.4	0	NA	OC
Karavasiloglou (2019) [45]	USA	522	All-cause mortality	14.5	27.5	47.1	Mixed
Blarigan (2019) [46]	USA	992	All-cause mortality	7	57	59.6	CC
Heitz (2018) [47]	USA	837	All-cause mortality Cancer-specific mortality	14	0	55.2	BC
Romaguera (2015) [48]	Europe	3292	All-cause mortality Cancer-specific mortality	4.2	45.5	64.6	CRC
Pelser(2014)(colon) [49]	USA	4213	All-cause mortality Cancer-specific mortality	5	68.1	68.4 +	CC
Pelser(2014)(rectal) [49]	USA	1514	All-cause mortality Cancer-specific mortality	5	70.9	68.3 +	RC
Inoue-Choi (2013) [21]	USA	2017	All-cause mortality Cancer-specific mortality CVD mortality	5.4	0	78.9	Mixed
Leger (2018) [50]	USA	2198	All-cause mortality CVD incidence	10.8	52	43.6	HCT survivors
Dixon (2023) [22]	USA and Canada	34,230	CVD mortality	29.1	56	6.0	Mixed

CRC: colorectal cancer; BC: breast cancer, PC: prostate cancer; OC: ovarian cancer, RC: rectal cancer, CC: colon cancer; HCT: hematopoietic cell transplantation

The main components included in the combined lifestyle score were diet, physical activity, body weight, cigarette smoking, and alcohol consumption (Table 2). Thirteen studies collected lifestyle information after cancer diagnosis, six studies collected before cancer diagnosis and three studies at both stages. Three major lifestyle scores were used: the World Cancer Research Fund/American Institute for Cancer Research (WCRF/AICR) score, the American Cancer Society (ACS) score, and a basic lifestyle score (Supplementary Table S5). When a study presented the results of two or more types of lifestyle scores, the results used in the synthesized estimates followed this sequence: WCRF/AICR score > ACS score > basic lifestyle score > other scores, considering their significance in practice [17].

**Table 2:** Components of healthy lifestyle score

First author(year)	Period of lifestyles assessment	Components of HLS							HLS	Comparisons
		BMI	Smoking	Drinking	Diet	PA	Sleep	BF		
Bian (2024) [11]	Post-diagnosis	+	+	+	+	+			Basic summing; total score:0–5	4–5 vs. 0–1
Barot (2024) [37]	Pre-diagnosis	+	+		+	+			Basic summing; total score: 0–4	4 vs. 0–1
Troeschel (2023) [12]	Post-diagnosis	+			+	+			Based on ACS/ASCO; total score: 0–18	11–18 vs. 0–7
Peng (2023) [13]	Post-diagnosis	+	+	+	+	+			Basic summing; total score: 0–5	4–5 vs. 0–1
Langlais (2023) [38]	Post-diagnosis	+		+	+	+			Based on WCRF/AICR; total score: 0–7 Based on ACS; total score: 0–4	Q3 vs. Q1
Ergas (2023) [39]	Post-diagnosis		+		+	+			Basic summing; total score:0–3	3 vs. 0
Cannioto (2023) [18]	Both	+	+	+	+	+			Based on AICR and ACS; total score: 0–7	Q4 vs. Q1
Liu (2022) [40]	Post-diagnosis	+	+	+	+	+			Basic summing; total score: 0–5	3–5 vs. 0–1
Graff (2022) [41]	Post-diagnosis	+		+	+	+			Based on WCRF/AICR; total score: 0–7 Based on ACS; total score: 0–6	WCRF/AICR: 5.25–7 vs. 0–2.5 ACS: 5–6 vs. 0–1.5
Zutphen (2021) [20]	Both	+		+	+	+			Based on WCRF/AICR; total score: 0–7 Based on ACS; total score: 0–8	WCRF/AICR: 4.5–7 vs. 0–2.5 ACS: 6–8 vs. 0–3
Sun (2021) [42]	Post-diagnosis	+	+	+		+	+		Basic summing; total score: 0–5	5 vs. 0–2
Song (2021) [19]	Both	+			+	+			Based on WCRF/AICR; total score: 0–3	Q4 vs. Q1
Cao (2021) [43]	Post-diagnosis		+	+	+	+	+		Basic summing; total score: 0–5	5 vs. 0–1
Minlikeeva (2019) [44]	Pre-diagnosis		+		+	+			Other methods	Never smoking/normal weight/active vs. Smoking/obesity/inactive
Karavasiloglou (2019) [45]	Post-diagnosis	+	+	+	+	+			Basic summing; total score: 0–5	3–5 vs. 0
Blarigan (2019) [46]	Post-diagnosis	+			+	+			Based on ACS; total score: 0–6	5–6 vs. 0–1
Heitz (2018) [47]	Pre-diagnosis	+	+	+	+	+			Other methods; total score: 0–12	8–12 vs. 0–3
Romaguera (2015) [48]	Pre-diagnosis	+		+	+	+		+	Based on WCRF/AICR; total score: 0–6 for men, 0–7 for women	men: 4–6 vs. 0–2 women: 5–7 vs. 0–3
Pelser (2014)(colon) [49]	Pre-diagnosis	+	+	+	+	+			Basic summing; total score: 0–5	5 vs. 0–1
Pelser (2014)(rectal) [49]	Pre-diagnosis	+	+	+	+	+			Basic summing; total score: 0–5	5 vs. 0–1
Inoue-Choi (2013) [21]	Post-diagnosis	+			+	+			Based on WCRF/AICR; total score: 0–8	6–8 vs. 0–4
Leger (2018) [50]	Pre-diagnosis		+		+	+			Basic summing; total score: 0–2	2–3 vs. 0
Dixon (2023) [22]	Post-diagnosis	+	+	+		+			Basic summing; total score: 0–4	3.5–4 vs. 0–2

HLS: healthy lifestyle score, BMI: body mass index, PA: physical activity, BF: breastfeeding; WCRF/AICR: World Cancer Research Fund/American Institute for Cancer Research, ACS: American Cancer Society, ACS/ASCO: American Cancer Society/American Society of Clinical Oncology

### Combined lifestyle factors and all-cause mortality

**Fig. 1:**
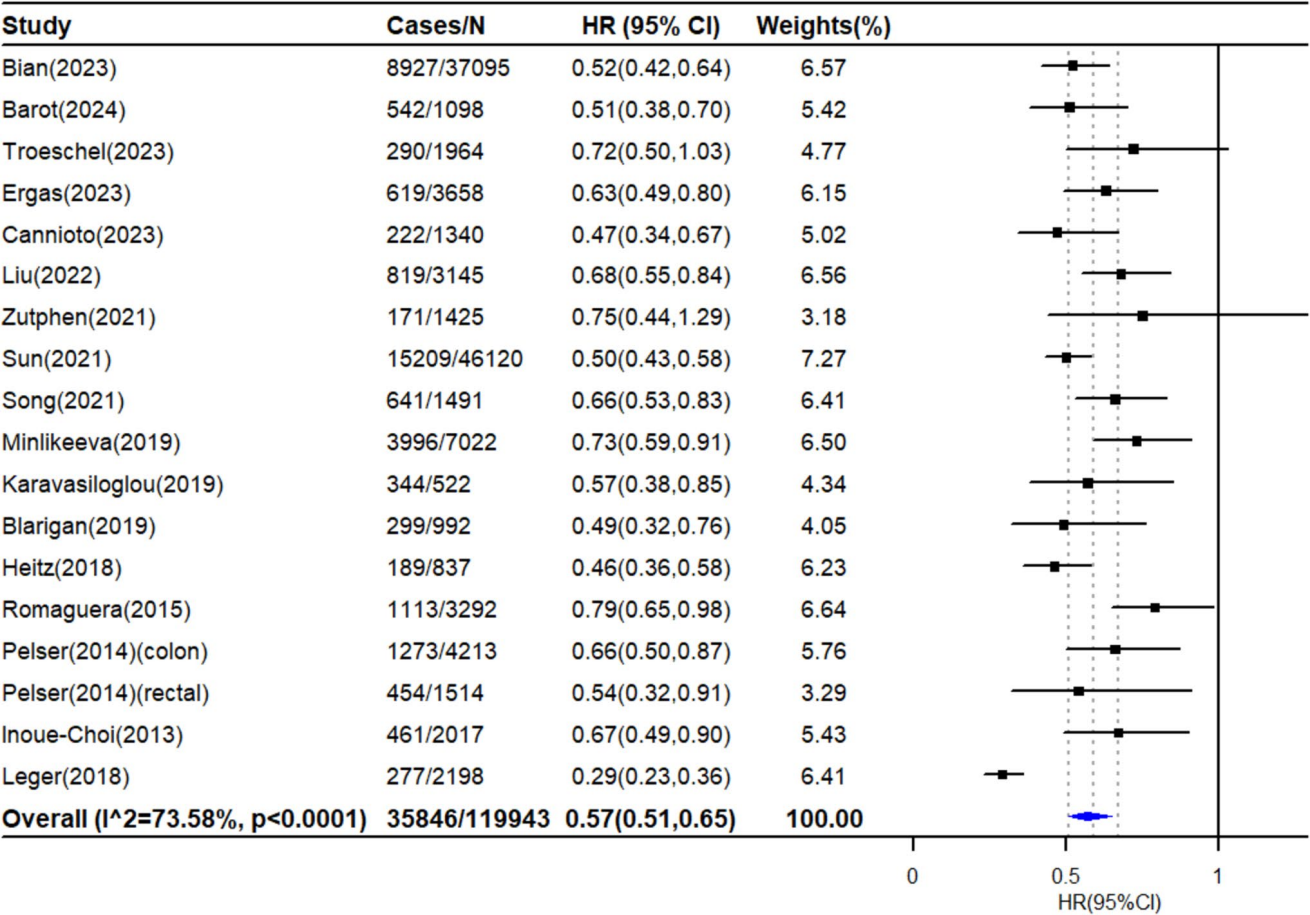
Association of combined lifestyle factors with all-cause mortality among cancer survivors. HR: hazard ratio, CI: confidence interval. The forest plot presented the HRs and 95%CI for comparing the risk of all-cause mortality between cancer survivors with the healthiest lifestyle pattern and those patients with the least healthy lifestyles. The HRs were represented by black squares, and CIs were represented by horizontal lines. The summary estimate is represented by the blue diamond. Estimates < 1.0 indicated protective association and HRs > 1.0 indicated an adverse relationship

**Fig. 2:**
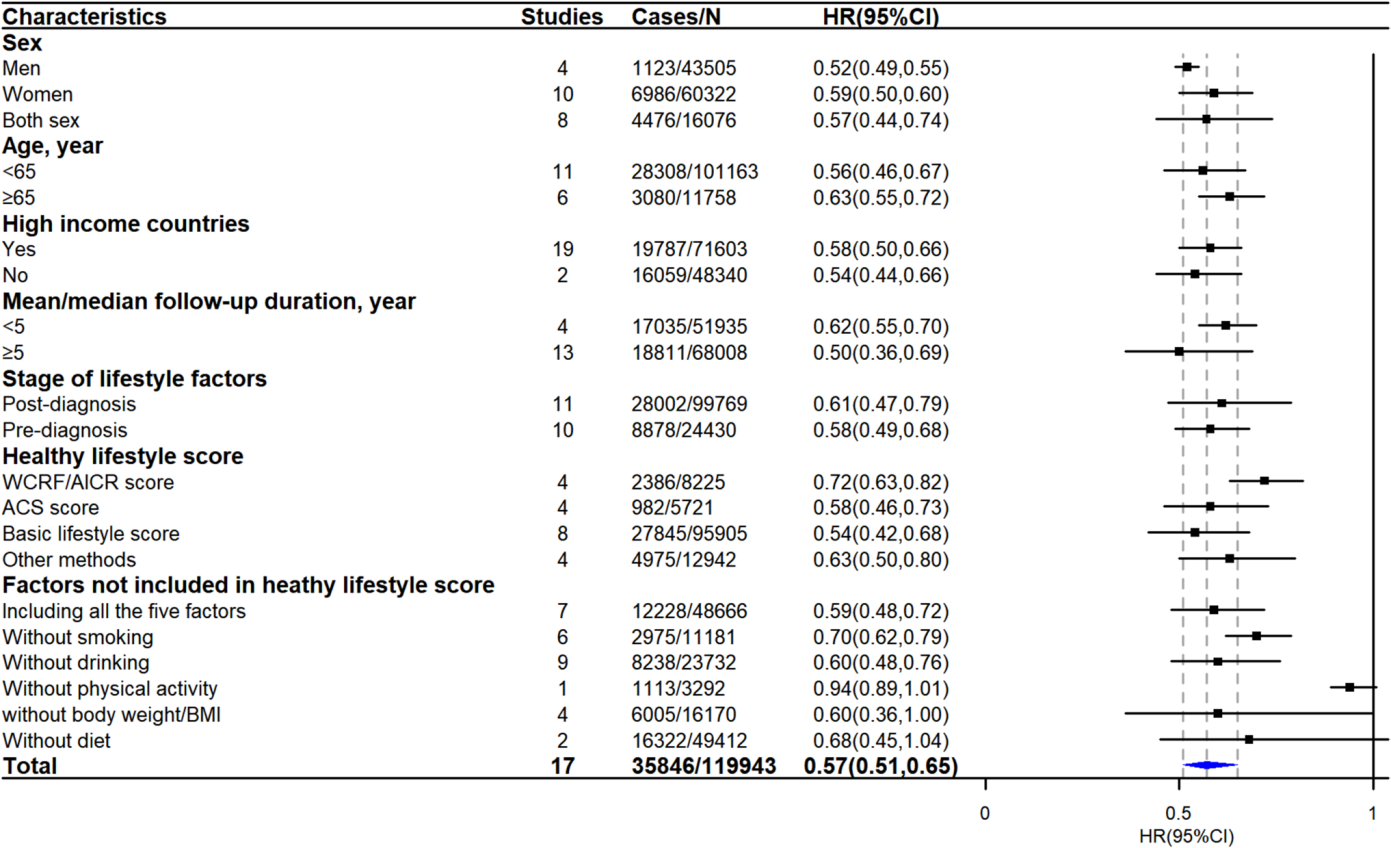
Subgroup meta-analysis for associations of combined lifestyle factors with all-cause mortality among cancer survivors. HR: hazard ratio, CI: confidence interval, WCRF/AICR: World Cancer Research Fund/American Institute for Cancer Research, ACS: American Cancer Society. The HRs were represented by black squares, and CIs were represented by horizontal lines. The summary estimate is represented by the blue diamond. Estimates < 1.0 indicated protective association and HRs > 1.0 indicated an adverse relationship

Seventeen studies reported results comparing participants with the healthiest lifestyle to those with the least healthy lifestyle for the risk of all-cause mortality, and the synthesized HR was 0.57 (95% CI: 0.51‒0.65, 119 943 survivors, Fig. 1). Substantial heterogeneity across studies was detected (*I^2* = 73.58%, *P* < 0.0001), which could be partly explained by the differences in study characteristics. As shown in the subgroup analyses (Fig. 2), the association between combined lifestyle factors and all-cause mortality decreased from 0.59 to 0.70 when the lifestyle score did not include smoking but included the other five main lifestyle factors. However, the results were largely similar in other subgroups. There were no obvious small study effects or publication bias according to the funnel plot (Supplementary Figure. S2) and Egger’s test (*P* = 0.78). The leave-one-out analysis revealed no evidence of strong influences from any individual studies on the pooled estimates (Supplementary Figure. S3). Seven studies reported results for the linear dose‒response meta-analysis (Supplementary Figure. S4). Consistent results were observed in the pooled analysis, with each one-unit increase in the combined healthy lifestyle score resulting in a 13% lower risk of all-cause mortality.

**Fig. 3:**
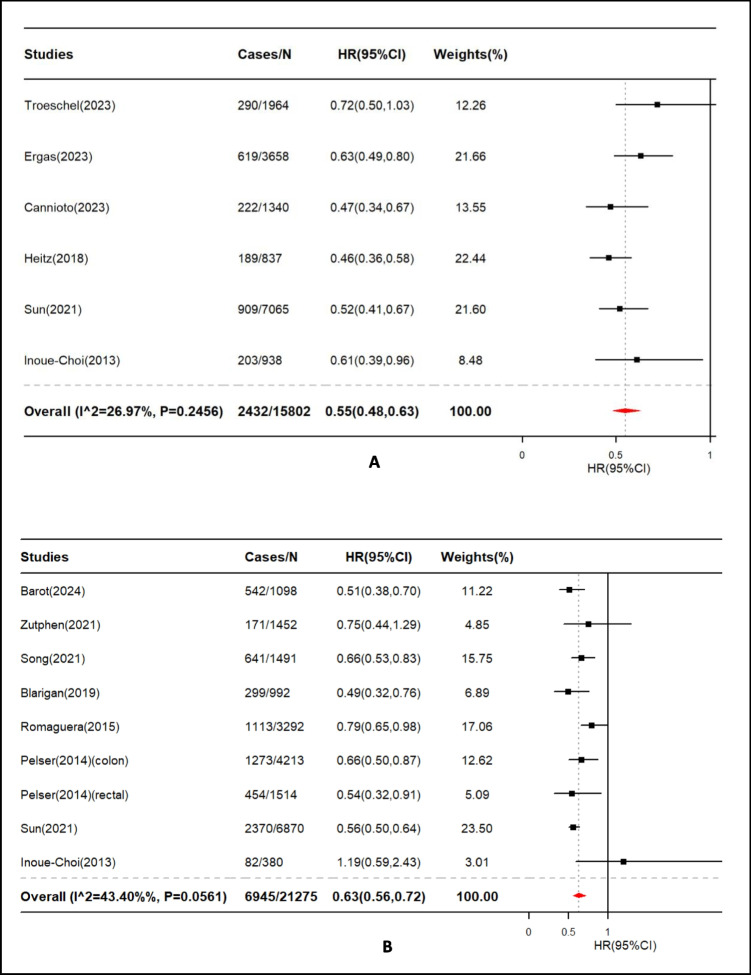
The association of combined lifestyle factors and all-cause mortality among breast (A) and colorectal (B) cancer survivors. HR: hazard ratio, CI: confidence interval. The HRs were represented by black squares, and CIs were represented by horizontal lines. The summary estimate is represented by the red diamonds. Estimates < 1.0 indicated protective association and HRs > 1.0 indicated an adverse relationship

We also found significant associations between combined lifestyle factors and all-cause mortality in breast cancer survivors (HR = 0.55, 95% CI: 0.48‒0.63, n = 6, 15 802 survivors, Fig. 3A) and colorectal cancer survivors (HR = 0.63, 95% CI: 0.56‒0.72, n = 8, 21 275 survivors, Fig. 3B). The relationships between lifestyles and all-cause mortality for survivors with a diagnosis of other cancers are displayed in Supplementary Figure. S5. A total of six studies that included data on eight cancer types are displayed. For survivors diagnosed with several cancer types (e.g., lung, liver, nasopharynx, gastric, kidney, and those treated with HCT), the data were obtained from a single study. No significant association was observed among prostate cancer survivors (HR = 0.80, 95% CI: 0.42‒1.51, n = 2). While a protective effect of combined lifestyles on all-cause mortality was detected among gynecologic cancer survivors (HR = 0.74, 95% CI: 0.60‒0.91, n = 2).

Three studies explored the relationship between changes in combined lifestyle factors and all-cause mortality among cancer survivors. Two of these studies found that a collective adherence to an overall healthier lifestyle is associated with significant mortality reductions [18, 19]. The third study reported that a 1-SD increase in the differences in the combined lifestyle score is related to a 16% lower all-cause mortality risk (HR = 0.84, 95% CI: 0.70‒0.99) [20].

### Combined lifestyle factors and cancer-specific mortality

**Fig. 4:**
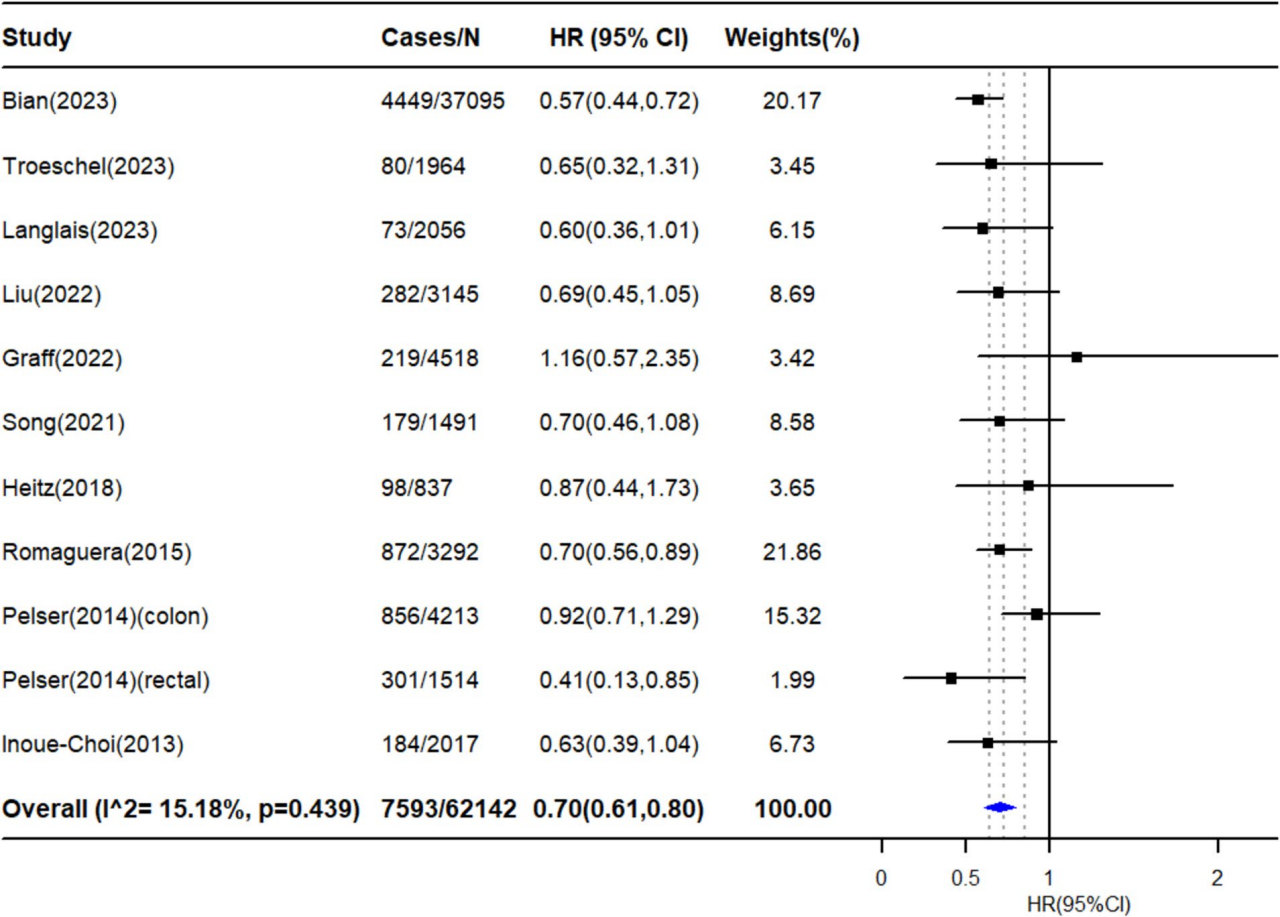
Associations of combined lifestyle and cancer-specific mortality among cancer survivors**.** HR: hazard ratio, CI: confidence interval. The forest plot presents the HRs (95%CI) comparing the risk of cancer-specific mortality between cancer survivors with the healthiest lifestyle pattern and those patients with the least healthy lifestyles. The HRs were represented by black squares, and CIs were represented by horizontal lines. The summary estimate is represented by the blue diamond. Estimates < 1.0 indicated protective association and HRs > 1.0 indicated an adverse relationship

Survivors with the healthiest lifestyle were more likely to have a 30% lower risk of cancer-specific mortality than those with the least healthy lifestyle 0.70 (95% CI: 0.61‒0.80, n = 10, 62 142 survivors, Fig. 4). No significant heterogeneity across studies was detected (*I^2* = 15.18%, *P* = 0.439). The results remained consistent in analyses stratified by sex, average age, period of lifestyles assessment, and average follow-up duration (Supplementary Figure. S6). There was no evidence of small study bias and publication bias (Egger’s test: *P* = 0.93). As suggested in the leave-one-out analysis, the summary results were not driven by a single study (Supplementary Figure. S7). Linear dose‒response analysis also yielded similar results (HR = 0.85, 95% CI: 0.79‒0.92, n = 7, 53 561 survivors) (Supplementary Figure. S8).

The results of subgroup analyses by cancer diagnosis are presented in Supplementary Figure. S9. Six studies covering three cancer sites were included. The results for gynecologic cancer survivors were from a single study. We detected a significant protective effect among colorectal cancer survivors (HR = 0.75, 95% CI: 0.63‒0.91). But no significant association was observed for breast cancer survivors.

### Combined lifestyle factors and cardiovascular disease

**Fig. 5:**
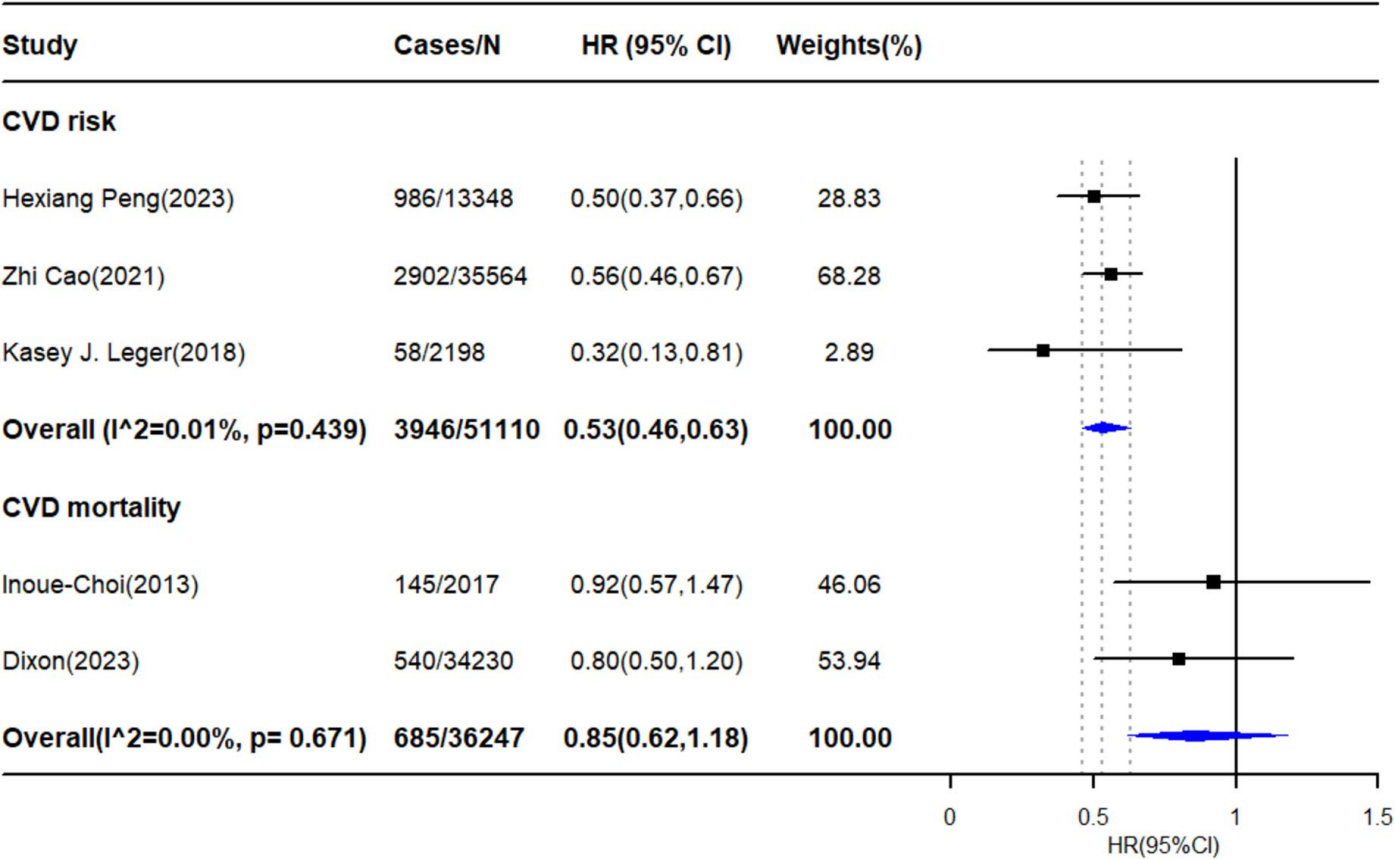
The association of combined lifestyle factors and cardiovascular disease among cancer survivors**.** HR: hazard ratio, CI: confidence interval. The forest plot presents the HRs (95%CI) comparing the risk of cardiovascular disease between cancer survivors with the healthiest lifestyle pattern and those patients with the least healthy lifestyles. The HRs were represented by black squares, and CIs were represented by horizontal lines. The summary estimate is represented by the blue diamond. Estimates < 1.0 indicated protective association and HRs > 1.0 indicated an adverse relationship

Three studies (51,110 cancer survivors) reported the results for CVD incidence and the summarized HR was 0.53 (95% CI: 0.46‒0.63, Fig. 5). No heterogeneity among the studies was observed (*I^2* = 0.01%, *P* = 0.44). There was no significant association for CVD‒specific mortality; however, only two cohorts were included in the pooled analysis. One study examined the effect of combined lifestyles on CVD‒specific mortality among older female cancer survivors, but null results were detected [21]. Similarly, non-significant associations between combined lifestyle factors and cardiac mortality were found among adult survivors of childhood cancer [22].

## Discussion

To the best of our knowledge, this is the most comprehensive and up-to-date systematic review and meta-analysis of cohort studies on the relationship between adherence to multiple healthy lifestyles and health outcomes among cancer survivors. By pooling the results of 22 studies, we found that a combination of healthier lifestyle factors was associated with the better prognosis of cancer survivors. Specifically, survivors with the healthiest lifestyle had 43%, 30%, and 47% lower risks of all-cause mortality, cancer-specific mortality and CVD incidence, respectively, compared with survivors with the least healthy lifestyles. These associations were largely consistent in analyses stratified by baseline characteristics and cancer type. However, no significant difference was detected for mortality due to CVD.

Although no meta-analyses or RCTs have explored the association between adherence to multiple healthy lifestyles and all-cause mortality among cancer survivors, the associations with individual lifestyle factors have been well established. For instance, meta-analyses have shown that increased intake of vegetables and fish is associated with decreased risk of overall mortality [23], and higher alcohol consumption and obesity are associated with increased total mortality in cancer survivors [23, 24]. Moreover, physical activity and smoking are also important lifestyle factors for the prognosis of cancer survivors. Evidence has shown that levels of physical activity are related to the risk of all-cause mortality in cancer survivors [25], and breast cancer survivors who currently smoke have a doubled risk of all-cause mortality [26]. In our stratified analysis, the associations decreased when physical activity or smoking were excluded separately from the overall lifestyle score, indicating the importance of physical activity and smoking for preventing premature death in cancer survivors. Furthermore, the large number of cancer survivors included in the current study allowed us to perform site-specific meta-analyses. The synthesized results, separate for breast and colorectal cancer, consistently showed that adopting a healthy lifestyle pattern had a protective effect on all-cause mortality. However, few studies have assessed such relationships among survivors living with or beyond other cancer types and more investigations are merited.

Apart from all-cause mortality, our meta-analysis revealed that cancer survivors with the healthiest lifestyle were likely to have a 30% lower risk of cancer-specific mortality, which is comparable to a previous review [7]. In that review (n = 3 studies, 6146 cancer survivors), survivors who had a healthy lifestyle reported a lower risk of cancer-specific mortality [7]. However, our study probably provided more robust findings because we further expanded the analysis by adding newly published evidence and pooling larger sample size (62,142 cancer survivors). This enabled us to conduct supplementary analyses to test the robustness of our findings (e.g., leave-one-out analyses). These relationships are largely consistent in cancer survivors of different sex, age groups, follow-up duration and periods of lifestyle factors assessment (pre- or post-diagnosis). This may have important implications for clinical and public health practice. For example, adopting a healthier lifestyle after the diagnosis of cancer is associated with a lower risk of cancer-specific mortality, which could provide evidence for health care professionals to encourage cancer survivors to change toward a healthier lifestyle.

CVDs are one of the most frequent adverse outcomes in cancer survivors [27], and they pose a greater mortality threat than cancer itself for some cancer types (e.g., breast cancer) [28]. In our study, we found that survivors with a healthy lifestyle have a reduced risk for CVD incidence. However, only three studies were included in the pooled analysis, and survivors in these studies were mainly breast cancer survivors from high-income countries. Nevertheless, our findings indicate that there is a potential to attenuate CVD risk in cancer survivors through adherence to healthy lifestyles. Only two studies from the USA reported the association between a combination of lifestyle factors and CVD mortality among cancer survivors. One study was carried out among 2017 female cancer survivors (145 events) [21], the other study was among 34,230 adult survivors of childhood cancers (504 events) [22], and no significant differences were detected. However, these findings might suffer from inadequate statistical power since a limited number of events occurred during the follow-up.

Our study has several strengths. It is the first systematic review and meta-analysis to investigate combined lifestyle factors and mortality in cancer survivors. The majority of the included studies were published recently (e.g., after 2020) and were generally of high-quality. In addition, a large number of cancer survivors with a minimum follow-up duration of 4.1 years, were included, which enabled us to conduct several supplementary analyses to show the robustness of our findings. Lastly, our study may have significant implications for clinical care, public health, and research. On the one hand, our findings suggest, that adherence to an overall healthy lifestyle is associated with lower risks for all-cause mortality, cancer-specific mortality, and CVD incidence in cancer survivors. These results could provide reliable evidence to clinical and public health professionals in guiding their efforts toward the long-term management of cancer survivors. Given that a large proportion of cancer survivors do not adopt the healthiest lifestyle [29, 30], interventions involving the modification of multiple lifestyles rather than an individual lifestyle, could be a public health priority. On the other hand, via systematic review methods, we were able to identify gaps in current research and provide directions for future investigations. Future studies with repeated measurements and focusing on survivors living with relatively rare cancer types, childhood cancer survivors, and survivors from low- and middle-income countries, are suggested.

Yet some limitations should also be acknowledged. First, there was heterogeneity across the studies due to differences in study characteristics (e.g., age, sex and cancer type), methodologies (e.g., analysis strategies, covariates included for adjustment), and definitions of healthy lifestyles. However, we performed leave-one-out analyses and found no evidence of a strong influence from any particular study on summary estimates. Additionally, our dose‒response meta-analysis produced similar results, suggesting that this heterogeneity is unlikely to substantially alter our overall conclusions. Second, most studies were conducted in high-income countries, which may limit the generalizability of our findings to other countries. Considering that lifestyles could greatly differ across countries [31, 32], more evidence from low- and middle-income countries is necessary. Third, few publications were available for CVD incidence in cancer survivors, restricting our full exploration for these outcomes. In addition, only three studies examined the associations between changes in combined lifestyles and all-cause mortality among cancer survivors with inconsistent methodology, more studies with repeated measurements are needed. Furthermore, there is increasing evidence that adult survivors of childhood cancer experience greater adverse outcomes and late mortality than their general counterparts due to the late effects of treatments, and that adopting healthier lifestyles might reduce the risk of late mortality [33, 34]. However, only one study on childhood cancer survivors is available, and more investigations are needed given the mounting burden of this population [35, 36]. Finally, although most studies adjusted for a wide range of confounders and we synthesized the results of the highest adjusted models, residual bias may still exist.

## Conclusion

In conclusion, we found that adherence to a healthy lifestyle is associated with a lower risk of all-cause mortality, cancer-specific mortality and CVD incidence in cancer survivors. Our findings indicate the potential benefits of changing to multiple healthier lifestyles in the long-term management of cancer survivors. More evidence among survivors living with rarer cancer diagnoses and from populations in low- and middle-income countries is still needed.

## Supplementary Information

Below is the link to the electronic supplementary material.

Supplementary file1 (DOCX 14751 KB)

## Data Availability

The datasets were derived from public sources and all data are incorporated into the article and its online supplementary material. The software code used in this study is available from the first author.
