# Supplementary material for: Combined lifestyle factors on mortality and cardiovascular disease among cancer survivors: a systematic review and meta-analysis of cohort studies
Source: Support Care Cancer. 2024 Dec 2;32(12):846. doi: 10.1007/s00520-024-09049-2 (PMC11611996; doi:10.1007/s00520-024-09049-2)
Supplement: Supplementary file 1 — Supplementary file1 (DOCX 14751 KB) [file 520_2024_9049_MOESM1_ESM.docx]

**Supplementary material**

**Combined lifestyle factors on mortality and cardiovascular disease among cancer survivors: a systematic review and meta-analysis of cohort studies**

**Supportive Care in Cancer**

Chunsu Zhu^1,2^, Zhiwei Lian ^1,2^, Volker Arndt^1^, Melissa S. Y. Thong^1*^

1. Unit of Cancer Survivorship, German Cancer Research Center, Heidelberg, Germany

2. Medical Faculty, University of Heidelberg, Heidelberg, Germany

**Corresponding author**

Melissa S. Y. Thong, Unit of Cancer Survivorship, German Cancer Research Center (DKFZ), Im Neuenheimer Feld 280, 69120, Heidelberg, Germany. Telephone: +49 6221 42-2334. Email: [m.thong@dkfz-heidelberg.de](mailto:m.thong@dkfz-heidelberg.de)

**Contents**

**Table S1**. Search strategy for PubMed

**Table S2**. Search strategy for Web of Science

**Table S3**. Search strategy for EMBASE

**Table S4.** Risk of bias assessed by the Newcastle-Ottawa Scale

**Table S5**. The definitions of the major lifestyle scores

**Figure. S1** The Flowchart of study selection.

**Figure. S2** Funnel plot for all-cause mortality

**Figure. S3** Influence analysis for all-cause mortality by excluding each individual study in turn

**Figure. S4** Dose-response meta-analysis for all-cause mortality

**Figure. S5** Umbrella review for all-cause mortality of cancer survivors other than survivors of breast and colorectal cancer

**Figure. S6** Subgroup meta-analysis for associations of combined lifestyle factors with cancer-specific mortality among cancer survivors

**Figure. S7** Influence analysis for cancer-specific mortality by excluding each individual study in turn

**Figure. S8** Dose-response meta-analysis for all-cause mortality

**Figure. S9** Umbrella review for cancer-specific mortality

**Table S1.** Search strategy for PubMed

| **Search** | **Query** |
| --- | --- |
| #1 | search(survivor*[Title/Abstract] OR patient*[Title/Abstract]) AND (cancer*[Title/Abstract] OR neoplasm[Title/Abstract] OR carcinoma[Title/Abstract]) |
| #2 | search(life style[Mesh] OR risk reduction behavior[Mesh] OR health behavior[Mesh] OR life style[Title/Abstract] OR life styles[Title/Abstract] OR "health factor"[Title/Abstract] OR "health factors"[Title/Abstract] OR lifestyle[Title/Abstract] OR lifestyles[Title/Abstract] OR "low risk"[Title/Abstract] OR "low risks"[Title/Abstract] OR prevention guideline[Title/Abstract] OR prevention guidelines[Title/Abstract] OR "protective factor"[Title/Abstract] OR "protective factors"[Title/Abstract] OR "risk reduction behavior"[Title/Abstract] OR "risk reduction behavior"[Title/Abstract] OR "risk reduction behaviors"[Title/Abstract] OR "risk reduction behaviors"[Title/Abstract] OR "health behavior"[Title/Abstract] OR "health behavior"[Title/Abstract] OR "health behaviors"[Title/Abstract] OR "health behaviors"[Title/Abstract] OR "healthy behavior"[Title/Abstract] OR "healthy behaviour"[Title/Abstract] OR "healthy behaviors"[Title/Abstract] OR "healthy behaviours"[Title/Abstract] OR "risk behavior"[Title/Abstract] OR "risk behaviour"[Title/Abstract] OR "risk behaviors"[Title/Abstract] OR "risk behaviours"[Title/Abstract] OR "modifiable factors"[Title/Abstract])) |
| #3 | search(combination[Title/Abstract] OR combinations[Title/Abstract] OR combined[Title/Abstract] OR composite[Title/Abstract] OR integrated[Title/Abstract] OR interaction[Title/Abstract] OR interactions[Title/Abstract] OR "joint effect"[Title/Abstract] OR "joint effects"[Title/Abstract] OR "merged effect"[Title/Abstract] OR "merged effects"[Title/Abstract] OR score[Title/Abstract] OR scores[Title/Abstract] OR "adherence to"[Title/Abstract] OR "adhere to"[Title/Abstract] OR "adhered to"[Title/Abstract] OR collective[Title/Abstract] OR cumulative[Title/Abstract] OR multiple[Title/Abstract] )) |
| #4 | search #2 AND#3 |
| #5 | search(cohort studies[Mesh] OR incidence[Mesh] OR survival analysis[Mesh] OR early diagnosis[Mesh] OR prognosis[Mesh] OR prospective[Title/Abstract] OR prospectively[Title/Abstract] OR cohort[Title/Abstract] OR follow up[Title/Abstract] OR "followed up"[Title/Abstract] OR longitudinal[Title/Abstract] OR "nested case-control"[Title/Abstract] OR "nested case control"[Title/Abstract] OR incidence[Title/Abstract] OR "early diagnosis"[Title/Abstract] OR predict[Title/Abstract] OR predicts[Title/Abstract] OR predicted[Title/Abstract] OR predicting[Title/Abstract] OR prediction[Title/Abstract] OR predictions[Title/Abstract] OR predictive[Title/Abstract] OR prognosis[Title/Abstract] OR prognoses[Title/Abstract] OR prognostic[Title/Abstract] OR survival[Title/Abstract]) |
| #6 | Search (death[Mesh] OR mortality[Mesh] OR death[Title/Abstract] OR deaths[Title/Abstract] OR fatal[Title/Abstract] OR fatality[Title/Abstract] OR fatalities[Title/Abstract] OR "life expectancy"[Title/Abstract] OR "life expectancies"[Title/Abstract] OR mortality[Title/Abstract] OR mortalities[Title/Abstract] OR survival[Title/Abstract] OR survive[Title/Abstract] OR survived[Title/Abstract] OR survives[Title/Abstract] OR surviving[Title/Abstract]) |
| #7 | Search (cardiovascular diseases[Mesh] OR cardiovascular[Title/Abstract] OR CVD[Title/Abstract] OR "heart disease"[Title/Abstract] OR "heart diseases"[Title/Abstract] OR "myocardial ischemia"[Title/Abstract] OR "myocardial ischemia"[Title/Abstract] OR AMI[Title/Abstract] OR IHD[Title/Abstract] OR CHD[Title/Abstract] OR "coronary artery disease"[Title/Abstract] OR "coronary artery diseases"[Title/Abstract] OR CAD[Title/Abstract] OR "myocardial infarction"[Title/Abstract] OR "heart infarction"[Title/Abstract] OR "acute coronary syndrome"[Title/Abstract] OR ACS[Title/Abstract] OR "heart failure"[Title/Abstract] OR "sudden cardiac death"[Title/Abstract] OR "cerebrovascular disorder"[Title/Abstract] OR "cerebrovascular disorders"[Title/Abstract] OR "cerebrovascular accident"[Title/Abstract] OR "cerebrovascular accidents"[Title/Abstract] OR "cerebrovascular attack"[Title/Abstract] OR "cerebrovascular attacks"[Title/Abstract] OR CVA[Title/Abstract] OR "cerebrovascular disease"[Title/Abstract] OR "cerebrovascular diseases"[Title/Abstract] OR CBVD[Title/Abstract] OR "cerebral arterial disease"[Title/Abstract] OR "cerebral arterial diseases"[Title/Abstract] OR stroke[Title/Abstract] OR strokes[Title/Abstract] OR transient ischemic attack[Title/Abstract] OR apoplex[Title/Abstract] OR apoplexy[Title/Abstract] OR “cardiac events” [Title/Abstract] OR “cardiac event” [Title/Abstract] OR “arteriosclerosis” [Title/Abstract] OR “atherosclerosis” [Title/Abstract] OR “brain ischemia” [Title/Abstract] OR “brain vascular accidents” [Title/Abstract] OR angina*[Title/Abstract] OR “cerebrovascular accident*”[Title/Abstract] OR cardiomyopathy[Title/Abstract] OR atherosclero*[Title/Abstract] OR “peripheral artery disease” [Title/Abstract] OR arteriosclero*[Title/Abstract]) |
| #8 | search #6 OR #7 |
| #9 | search #1 AND #4 AND #5 AND #8 |

**Table S2**. Search strategy for Web of Science

| **Search** | **Query** |
| --- | --- |
| #1 | (AB=(survivor* OR patient* )) AND AB=(cancer OR neoplasm OR carcinoma) |
| #2 | #3 AND #4 |
| #3 | AB=( life style OR risk reduction behavior OR health behavior OR life style OR life styles OR "health factor" OR "health factors" OR lifestyle OR lifestyles OR "low risk" OR "low risks" OR prevention guideline OR prevention guidelines OR "protective factor" OR "protective factors" OR "risk reduction behavior" OR "risk reduction behaviour" OR "risk reduction behaviors" OR "risk reduction behaviours" OR "health behavior" OR "health behaviour" OR "health behaviors" OR "health behaviours" OR "healthy behavior" OR "healthy behaviour" OR "healthy behaviors" OR "healthy behaviours" OR "risk behavior" OR "risk behaviour" OR "risk behaviors" OR "risk behaviours" OR "modifiable factors") |
| #4 | AB=(combination OR combinations OR combined OR composite OR integrated OR interaction OR interactions OR "joint effect" OR "joint effects" OR "merged effect" OR "merged effects" OR score OR scores OR "adherence to" OR "adhere to" OR "adhered to" OR collective OR cumulative OR multiple) |
| #5 | #6 OR #7 |
| #6 | AB=(death OR mortality OR death OR deaths OR fatal OR fatality OR fatalities OR "life expectancy" OR "life expectancies" OR mortality OR mortalities OR survival OR survive OR survived OR survives OR surviving) |
| #7 | AB=(cardiovascular diseases OR cardiovascular OR CVD OR "heart disease" OR "heart diseases" OR "myocardial ischemia" OR "myocardial ischemia" OR AMI OR IHD OR CHD OR "coronary artery disease" OR "coronary artery diseases" OR CAD OR "myocardial infarction" OR "heart infarction" OR "acute coronary syndrome" OR ACS OR "heart failure" OR "sudden cardiac death" OR "cerebrovascular disorder" OR "cerebrovascular disorders" OR "cerebrovascular accident" OR "cerebrovascular accidents" OR "cerebrovascular attack" OR "cerebrovascular attacks" OR CVA OR "cerebrovascular disease" OR "cerebrovascular diseases" OR CBVD OR "cerebral arterial disease" OR "cerebral arterial diseases" OR stroke OR strokes OR apoplexy OR “cardiac events” OR “cardiac event” OR “arteriosclerosis” OR “atherosclerosis” OR “brain ischemia” OR “brain vascular accidents” OR angina* OR “cerebrovascular accident*” OR cardiomyopathy OR atherosclero* OR “peripheral artery disease” OR arteriosclero*) |
| #8 | AB=(cohort studies OR incidence OR survival analysis OR early diagnosis OR prognosis OR prospective OR prospectively OR cohort OR follow up OR "followed up" OR longitudinal OR "nested case-control" OR "nested case control" OR incidence OR "early diagnosis" OR predict OR predicts OR predicted OR predicting OR prediction OR predictions OR predictive OR prognosis OR prognoses OR prognostic OR survival) |
| #9 | #8 AND #5 AND #2 AND #1 |

**Table S3**. Search strategy for EMBASE

| **Search** | **Query** |
| --- | --- |
| #1 | Search((survivor*:ab,ti OR patient*:ab,ti) AND (cancer:ab,ti OR neoplasm:ab,ti OR carcinoma:ab,ti)) |
| #2 | search(('life style':ab,ti OR 'life styles':ab,ti OR 'health factor':ab,ti OR 'health factors':ab,ti OR lifestyle:ab,ti OR lifestyles:ab,ti OR 'low risk':ab,ti OR 'low risks':ab,ti OR 'prevention guideline':ab,ti OR 'prevention guidelines':ab,ti OR 'protective factor':ab,ti OR 'protective factors':ab,ti OR 'risk reduction behavior':ab,ti OR 'risk reduction behaviour':ab,ti OR 'risk reduction behaviors':ab,ti OR 'risk reduction behaviours':ab,ti OR 'health behavior':ab,ti OR 'health behaviour':ab,ti OR 'health behaviors':ab,ti OR 'health behaviours':ab,ti OR 'healthy behavior':ab,ti OR 'healthy behaviour':ab,ti OR 'healthy behaviors':ab,ti OR 'healthy behaviours':ab,ti OR 'risk behavior':ab,ti OR 'risk behaviour':ab,ti OR 'risk behaviors':ab,ti OR 'risk behaviours':ab,ti OR 'modifiable factors':ab,ti) AND (combination:ab,ti OR combinations:ab,ti OR combined:ab,ti OR composite:ab,ti OR integrated:ab,ti OR interaction:ab,ti OR interactions:ab,ti OR 'joint effect':ab,ti OR 'additive effect':ab,ti OR 'joint effects':ab,ti OR 'merged effect':ab,ti OR 'merged effects':ab,ti OR score:ab,ti OR scores:ab,ti OR 'adherence to':ab,ti OR 'adhere to':ab,ti OR 'adhered to':ab,ti OR collective:ab,ti OR cumulative:ab,ti OR multiple:ab,ti)) |
| #3 | search(death:ab,ti OR deaths:ab,ti OR fatal:ab,ti OR fatality:ab,ti OR fatalities:ab,ti OR 'life expectancy':ab,ti OR 'life expectancies':ab,ti OR mortality:ab,ti OR mortalities:ab,ti OR survival:ab,ti OR survive:ab,ti OR survived:ab,ti OR survives:ab,ti OR surviving:ab,ti) |
| #4 | search('cardiovascular diseases':ab,ti OR cardiovascular:ab,ti OR cvd:ab,ti OR 'heart disease':ab,ti OR 'heart diseases':ab,ti OR 'myocardial ischemia':ab,ti OR ami:ab,ti OR ihd:ab,ti OR chd:ab,ti OR 'coronary artery disease':ab,ti OR 'coronary artery diseases':ab,ti OR cad:ab,ti OR 'myocardial infarction':ab,ti OR 'heart infarction':ab,ti OR 'acute coronary syndrome':ab,ti OR acs:ab,ti OR 'heart failure':ab,ti OR 'sudden cardiac death':ab,ti OR 'cerebrovascular disorder':ab,ti OR 'cerebrovascular disorders':ab,ti OR 'cerebrovascular accident':ab,ti OR 'cerebrovascular accidents':ab,ti OR 'cerebrovascular attack':ab,ti OR 'cerebrovascular attacks':ab,ti OR cva:ab,ti OR 'cerebrovascular disease':ab,ti OR 'cerebrovascular diseases':ab,ti OR cbvd:ab,ti OR 'cerebral arterial disease':ab,ti OR 'cerebral arterial diseases':ab,ti OR stroke:ab,ti OR strokes:ab,ti OR apoplex:ab,ti OR 'cardiac events':ab,ti OR 'cardiac event':ab,ti OR ' arteriosclerosis ':ab,ti OR ' atherosclerosis ':ab,ti OR ' brain ischemia ':ab,ti OR ' brain vascular accidents ':ab,ti OR ' angina* ':ab,ti OR ' cerebrovascular accident* ':ab,ti OR ' cardiomyopathy ':ab,ti OR ' atherosclero* ':ab,ti OR ' peripheral artery disease ':ab,ti OR ‘arteriosclero*’ :ab,ti) |
| #5 | search #3 or #4 |
| #6 | search('cohort studies':ab,ti OR 'survival analysis':ab,ti OR prospective:ab,ti OR prospectively:ab,ti OR cohort:ab,ti OR 'follow up':ab,ti OR 'followed up':ab,ti OR longitudinal:ab,ti OR 'nested case-control':ab,ti OR 'nested case control':ab,ti OR incidence:ab,ti OR 'early diagnosis':ab,ti OR predict:ab,ti OR predicts:ab,ti OR predicted:ab,ti OR predicting:ab,ti OR prediction:ab,ti OR predictions:ab,ti OR predictive:ab,ti OR prognosis:ab,ti OR prognoses:ab,ti OR prognostic:ab,ti OR survival:ab,ti) |
| #7 | search #6 AND #5 AND #2 AND #1 |

**Table S4**. Risk of bias assessed by the Newcastle-Ottawa Scale

|  | **Selection of cohorts** | | | | **Comparability of cohorts** | | | **Assessment of outcome** | | | **Total score** |
| --- | --- | --- | --- | --- | --- | --- | --- | --- | --- | --- | --- |
| **Author(year)** | **REC** | **SNEC** | **AE** | **DNO** | **Demographic characteristics** | **Health status** | **Other factors** | **FULE** | **AO** | **AFUC** |  |
| Bian(2023) | 1 | 1 | 1 | 1 | 1 | 1 | 1 | 1 | 1 | 0 | 9 |
| Barot(2024) | 1 | 1 | 1 | 1 | 1 | 0 | 0 | 0 | 1 | 0 | 6 |
| Troeschel(2023) | 1 | 1 | 1 | 1 | 1 | 1 | 1 | 1 | 1 | 1 | 9 |
| Peng(2023) | 1 | 1 | 1 | 1 | 1 | 1 | 1 | 1 | 1 | 0 | 9 |
| Langlais(2023) | 1 | 1 | 1 | 1 | 1 | 1 | 1 | 1 | 1 | 0 | 9 |
| Ergas(2023) | 1 | 1 | 1 | 1 | 1 | 1 | 1 | 1 | 1 | 1 | 9 |
| Cannioto(2023) | 1 | 1 | 1 | 1 | 1 | 1 | 1 | 1 | 1 | 0 | 9 |
| Liu(2022) | 1 | 1 | 1 | 1 | 1 | 1 | 1 | 1 | 1 | 0 | 9 |
| Graff(2022) | 1 | 1 | 1 | 1 | 1 | 0 | 1 | 1 | 1 | 1 | 9 |
| Zutphen(2021) | 1 | 1 | 1 | 1 | 1 | 1 | 1 | 0 | 1 | 0 | 8 |
| Sun(2021) | 1 | 1 | 1 | 1 | 1 | 0 | 1 | 0 | 1 | 0 | 7 |
| Song(2021) | 1 | 1 | 1 | 1 | 1 | 1 | 1 | 1 | 1 | 0 | 9 |
| Cao(2021) | 1 | 1 | 1 | 1 | 1 | 1 | 1 | 1 | 1 | 1 | 10 |
| Minlikeeva(2019) | 1 | 1 | 0 | 1 | 1 | 0 | 1 | 1 | 0 | 0 | 6 |
| Karavasiloglou(2019) | 0 | 1 | 1 | 1 | 1 | 0 | 1 | 1 | 1 | 1 | 8 |
| Blarigan(2019) | 0 | 1 | 1 | 1 | 1 | 1 | 1 | 1 | 1 | 0 | 8 |
| Heitz(2018) | 0 | 1 | 1 | 1 | 1 | 1 | 1 | 1 | 1 | 1 | 9 |
| Romaguera(2015) | 1 | 1 | 1 | 1 | 1 | 1 | 1 | 0 | 1 | 0 | 8 |
| Pelser(2014) | 1 | 1 | 1 | 1 | 1 | 1 | 1 | 1 | 1 | 0 | 9 |
| Inoue-Choi(2013) | 1 | 1 | 1 | 1 | 1 | 1 | 1 | 1 | 1 | 0 | 9 |
| Leger(2018) | 1 | 1 | 0 | 1 | 1 | 1 | 1 | 1 | 0 | 1 | 8 |
| Dixon (2023) | 1 | 1 | 1 | 1 | 1 | 1 | 1 | 1 | 1 | 1 | 10 |

REC: Representativeness of exposed cohorts. SNEC: selection of the non-exposed cohorts. AE: DNO: Demonstration of outcomes of interests are not presented at the start of studies. FULE: follow-up duration was long enough for outcome to occur. AO: assessment of outcome. AFUC: the follow-up period was adequate for cohort. Ascertainment of exposure, according to guideline or basic summing is assigned to 1 score, other methods 0. Comparability of cohorts were assessed by whether the model adjusted for the following covariates, demographic characteristics (age, sex, education, income, or marital status), health status (physical comorbidities, stage of cancer) and other factors.

The follow-up duration was not regarded as long enough for the outcome to occur if the mean follow-up duration was less than 5 Years.

If the follow-up duration was not reported or if it is less than 10 years, then the follow-up duration will not be deemed as adequate follow-up duration.

AO is given 1 point if outcomes were obtained from objective sources, such as medical records or regional death registration system, if the methods for the obtaining of outcomes were not reported, the AO was recorded as 0 score.

**Table S5.** The definitions of the major lifestyle scores

| Lifestyle factors | Basic summing of lifestyle score | WCRF/AICR score | ACS score |
| --- | --- | --- | --- |
| Smoking | 1 point: never smokers; 0 point: ever smokers. | Not included | Not included |
| Drinking | 1 point: light drinkers (<28 g/day for men and <14 g/day for women); 0 point: other drinkers | 1 point: 0g/day; 0.5 point: men(0-20g/day), women (<10g/day); 0: men (>20g/day), women (>10 g/day). | 2 points: men (0-20 g/day), women (<10g/day); 1 point: 0g/day; 0 point: men (>20 g/day), women (>10g/day). |
| Physical activity | 1 point: moderate-to-vigorous leisure-time physical activity (≥150 min/week) or ≥150 min of moderate activity per week, or ≥75 min of vigorous activity per week; 0 point: for others. | 1: ≥ 150min/week moderate or vigorous physical activity; 0.5: 75-150min/week moderate or vigorous physical activity; 0: < 75min/week moderate or vigorous physical activity. | 2 point: ≥ 300min/week moderate or vigorous physical activity; 1 point: 150-300min/week moderate or vigorous physical activity; 0 point: < 150min/week moderate or vigorous physical activity. |
| Body mass index (kg/m^2^) | 1 point: optimal BMI (18.5–24.9); 0: others. | 1 point: 18.5-25; 0.5 point: for 25-30; 0 point: ≥30. | 2 point: 18.5-25; 1 point: for 25-30; 0 point: ≥30. |
| Diet | Measured by Healthy Eating Index (HEI): 1 point: HEI at the highest tertile; 0 point: others. Or adequate intake of dietary components including fruits, vegetables, whole grains, refined grains, fish, unprocessed meat, and processed meat: 1 point: ≥4 types; 0 point: <3 types. | Not included | Diet score was calculated by summing the score of the following three dietary components (red and processed meat, whole fruits and vegetables, and total grains):  2 points: diet score 7-9; 1 point: diet score 3-6; 0 point: diet score 0-2. |
| Red and processed meat | Not included. | 1 point: red meat < 500g/week and processed meat<21g/week; 0.5 point: red meat <500g/week and processed meat 21-100g/week; 0 point: red meat ≥500g/week or ≥100g/week processed meat. | 3 points for first quartile; 2 points for the second quartile; 1 point for the third quartile; 0 point for the forth quartile. |
| Whole fruits and vegetables | Not included. | 1 point: ≥ 400g/day; 0.5 point: 200-400g/day; 0 point: < 200g/day. | 2 points: the third tertile of unique fruits and vegetables/month; 1 point: the second tertile of unique fruits and vegetables/month; 0 point: the first tertile of unique fruits and vegetables/month. |
| Total grains | Not included. | Not included | 3 points: the fourth quartile; 2 points: the third quartile; 1 point: the second quartile; 0 point: the first quartile. |
| Ultra-processed foods |  | 1 point: tertile1; 0.5 point: tertile2; 0 point: tertile3. | Not included. |
| Fiber | Not included. | 1 point: ≥30g/day; 0.5 point: 15-30g/day; 0 point: < 15/day. | Not included. |
| Sugar-sweetened beverages | Not included. | 1 point: 0g/day; 0.5 point: 0-250g/day; 0 point: > 250g/day. | Not included. |

**Figure S1**. The Flowchart of study selection. CVD: cardiovascular disease.

32,005 articles identified though database searches

EMBASE: n=16,868

PubMed: n=7628

Web of Science: n=7509

Excluding duplicates: n=13,391

18,614 titles and abstracts screened

40 excluded

23 examined individual lifestyle factor or a combination of only two lifestyle factors

12 unrelated to pre-decided outcomes, cancer survivors, or cohort studies

5 duplicated

Full-text screening: n= 62

22 studies included in analyses

17 reported all-cause mortality

11 reported cancer-specified mortality

3 reported CVD incidence

2 reported CVD mortality

18,563 excluded

1715 unrelated to cancer survivors

13,606 unrelated exposures or pre-decided outcomes

3242 not prospective cohort studies

11 articles from relevant original studies and reviews


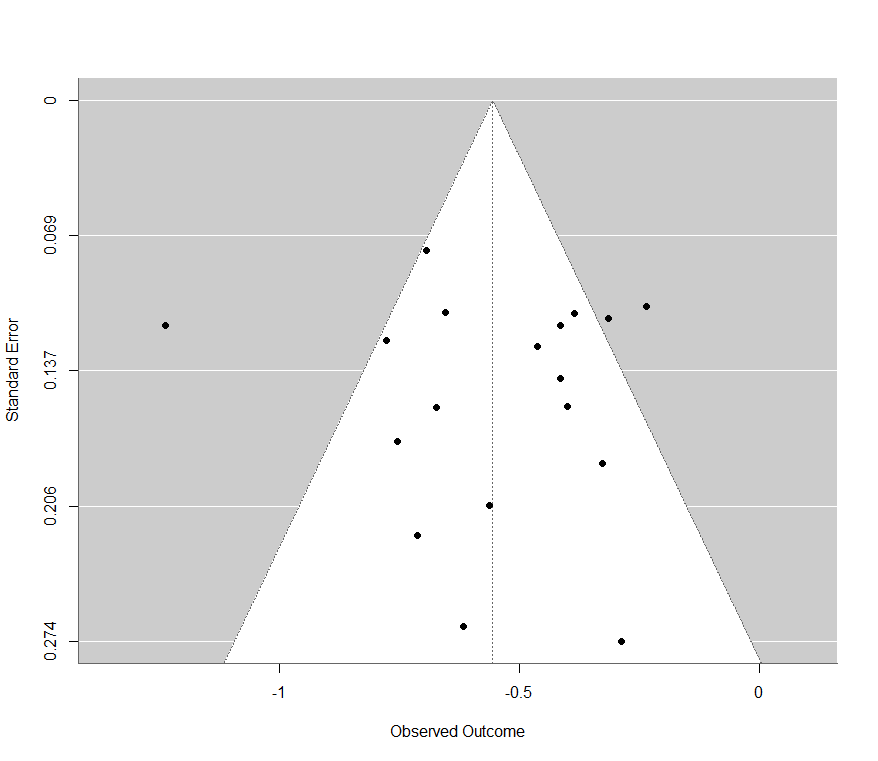
**Figure. S2** Funnel plot for all-cause mortality


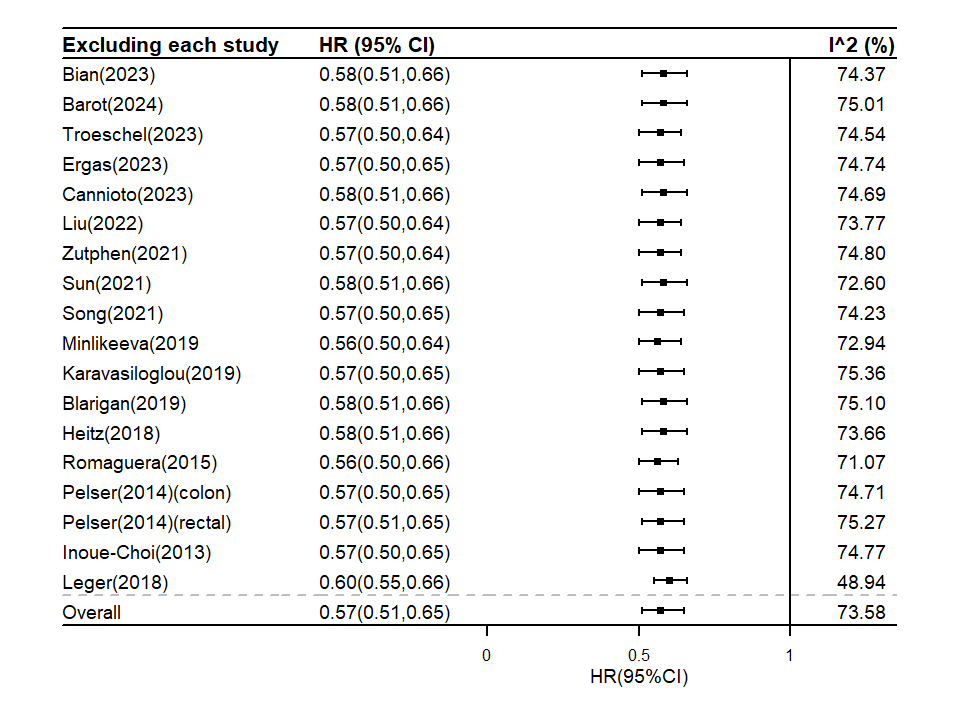


**Figure. S3** Influence analysis for all-cause mortality by excluding each individual study in separately

HR: hazard ratio, CI: confidence interval. The HRs were represented by black squares, and CIs were represented by horizontal lines. Estimates < 1.0 indicated protective association and HRs > 1.0 indicated an adverse relationship.


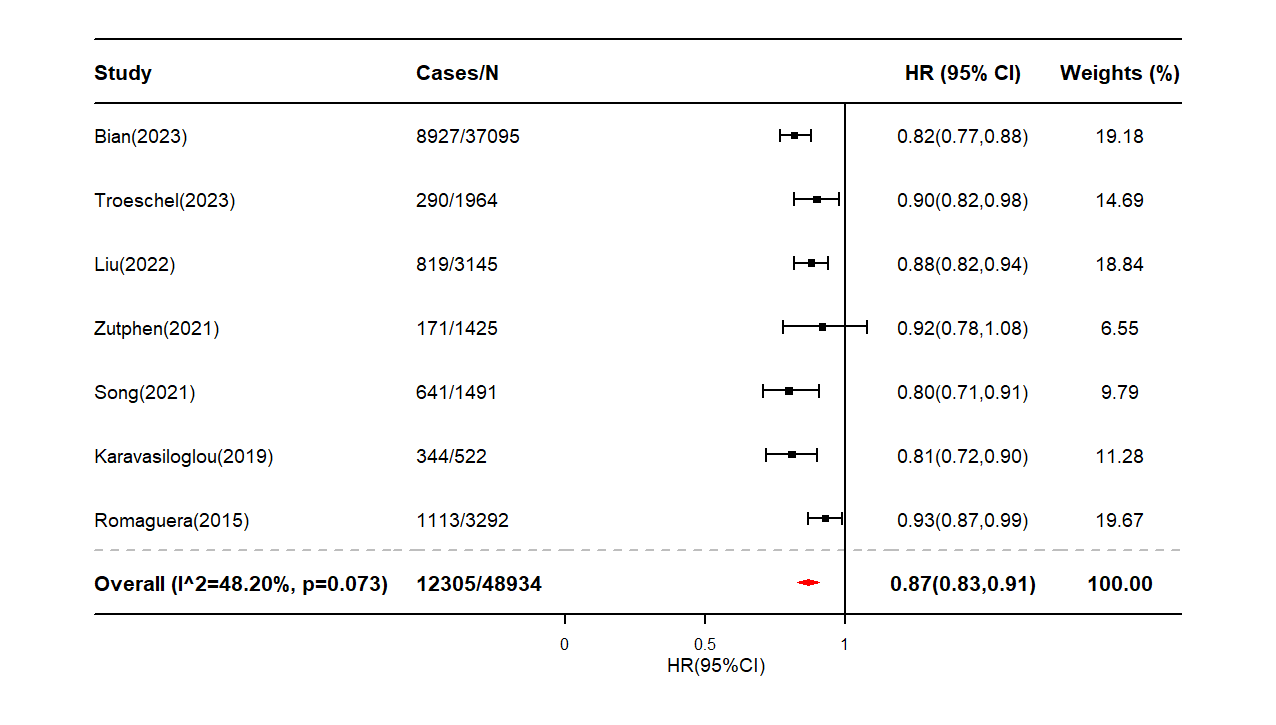


**Figure. S4** Dose-response meta-analysis for all-cause mortality

HR: hazard ratio, CI: confidence interval. The forest plot shows the risk of all-cause mortality with per unit increase in the combined lifestyle scores. The HRs were represented by black squares, and CIs were represented by horizontal lines. The summary estimate is represented by the red diamond. Estimates < 1.0 indicated protective association and HRs > 1.0 indicated an adverse relationship.


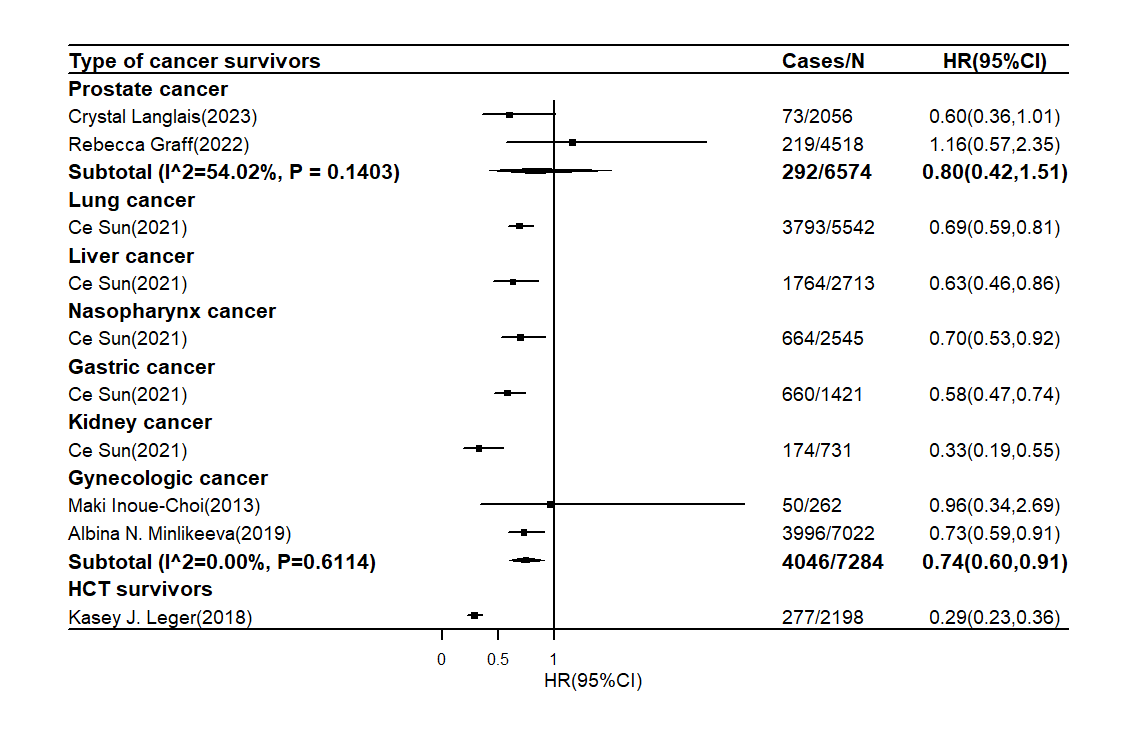


**Figure. S5** Umbrella review for all-cause mortality of cancer survivors other than survivors of breast and colorectal cancer

HR: hazard ratio, CI: confidence interval, HCT: hematopoietic cell transplantation. The HRs were represented by black squares, and CIs were represented by horizontal lines. The summary estimate is represented by the black diamond. Estimates < 1.0 indicated protective association and HRs > 1.0 indicated an adverse relationship.


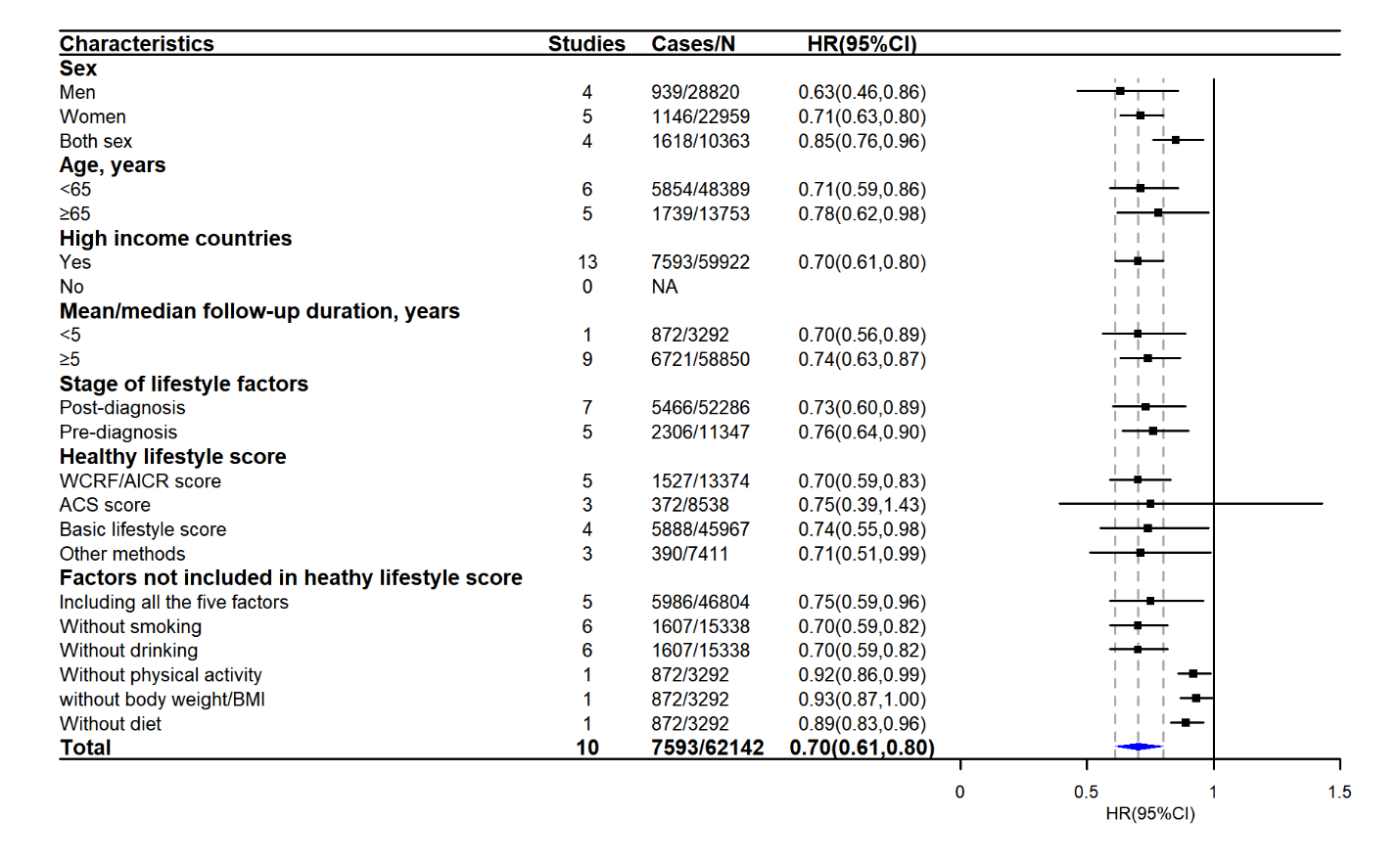


**Figure. S6** Subgroup meta-analysis for associations of combined lifestyle factors with cancer-specific mortality among cancer survivors

HR: hazard ratio, CI: confidence interval, WCRF/AICR: World Cancer Research Fund/American Institute for Cancer Research, ACS: American Cancer Society. The HRs were represented by black squares, and CIs were represented by horizontal lines. The summary estimate is represented by the blue diamond. Estimates < 1.0 indicated protective association and HRs > 1.0 indicated an adverse relationship.


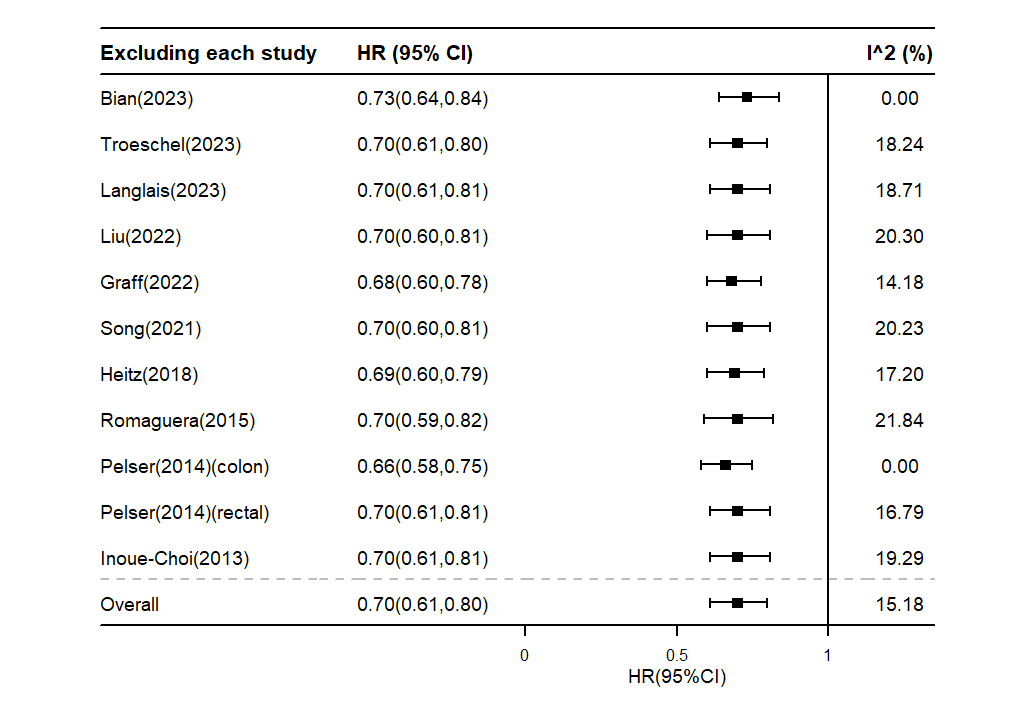


**Figure. S7** Influence analysis for cancer-specific mortality by excluding each individual study in turn

HR: hazard ratio, CI: confidence interval. The HRs were represented by black squares, and CIs were represented by horizontal lines. Estimates < 1.0 indicated protective association and HRs > 1.0 indicated an adverse relationship.


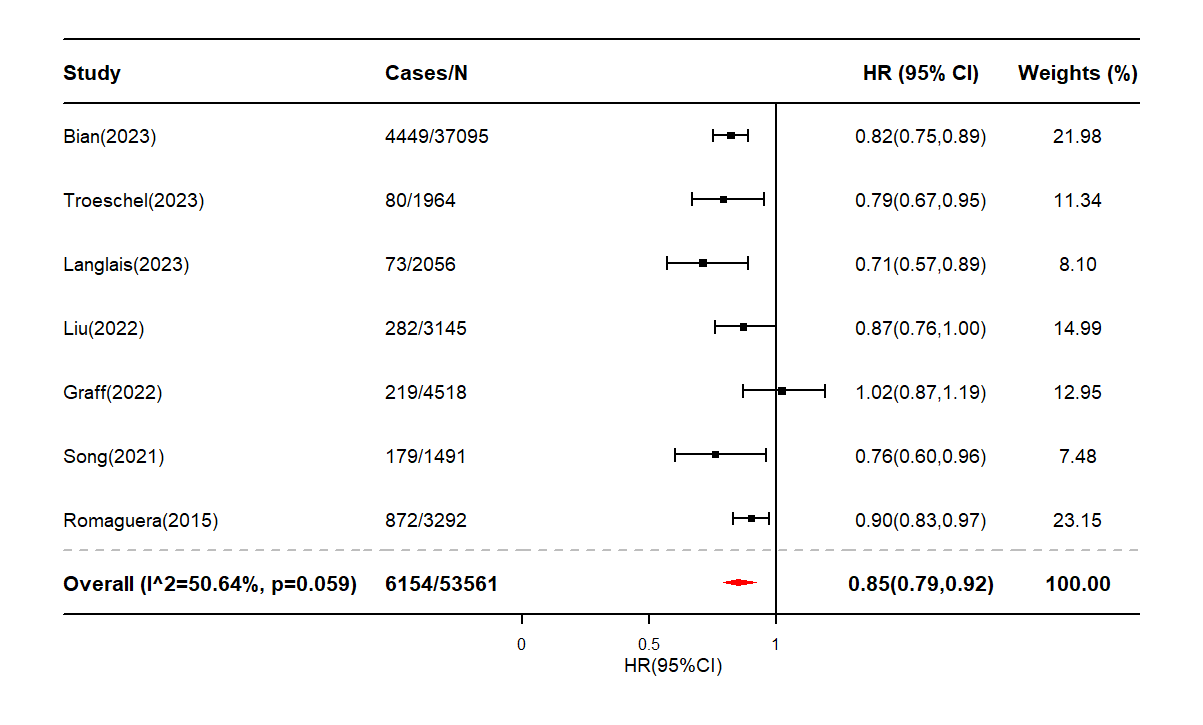


**Figure. S8** Dose-response meta-analysis for cancer-specific mortality

HR: hazard ratio, CI: confidence interval. The forest plot shows the risk of cancer-specific mortality with per unit increase in the combined lifestyle scores. The HRs were represented by black squares, and CIs were represented by horizontal lines. The summary estimate is represented by the red diamond. Estimates < 1.0 indicated protective association and HRs > 1.0 indicated an adverse relationship.


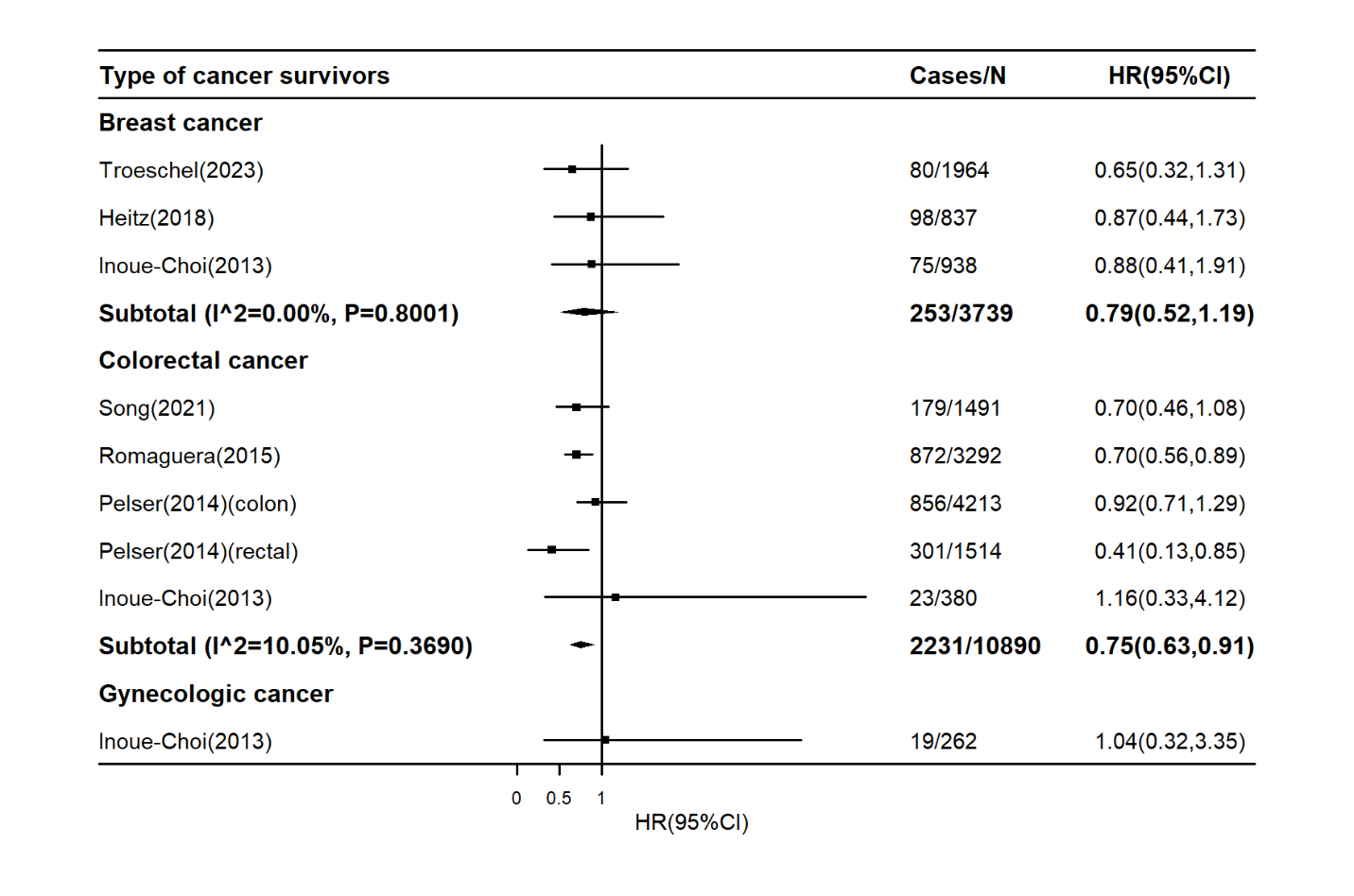


**Figure. S9** Umbrella review for cancer-specific mortality of survivors with specific cancer diagnosis

HR: hazard ratio, CI: confidence interval. The HRs were represented by black squares, and CIs were represented by horizontal lines. The summary estimate is represented by the black diamond. Estimates < 1.0 indicated protective association and HRs > 1.0 indicated an adverse relationship.
